# Rapid detection of non-rifampicin resistant drug-resistant tuberculosis using Xpert MTB/XDR testing: Findings from a multisite drug-resistant tuberculosis case detection surge in Zambia

**DOI:** 10.1371/journal.pgph.0005536

**Published:** 2026-09-23

**Authors:** Phallon Blessing Mwaba, Marshal Sikandangwa, Robertson Chibumbya, Innocent Mwaba, Minyoi Mubita Maimbolwa, Chitalu Chanda, Muhammad Mputu, Rhehab Chimzizi, Kevin Mabvuto Zimba, Nancy Kasese Chanda, Angel Mubanga, Monde Muyoyeta, Mary Kagujje

**Affiliations:** 1 Department of Tuberculosis and other Respiratory Diseases, Centre for Infectious Disease Research in Zambia, Lusaka, Zambia; 2 Department of Public Health, Ministry of Health, Zambia, National Tuberculosis and Leprosy Programme, Lusaka, Zambia; 3 Health Coordination Office, United States Embassy, Lusaka, Zambia; PLOS: Public Library of Science, UNITED STATES OF AMERICA

## Abstract

Zambia continues to face a high-Tuberculosis burden with rising drug resistance, yet diagnosis of non-rifampicin resistant drug-resistance remains limited due to reliance on rifampicin as a proxy for multidrug-resistant tuberculosis. The Xpert MTB/XDR assay offers a rapid detection of resistance to isoniazid, fluoroquinolone, Ethionamide and aminoglycosides, addressing a major diagnostic gap. The aim of this analysis was to describe the prevalence and profile of drug resistance among bacteriologically confirmed TB patients subjected to reflex Xpert MTB/XDR testing. We conducted an analysis of programmatic data from a four-week active drug-resistant TB case finding surge in May 2025, implemented across 39 high-TB burden facilities in five provinces of Zambia. We re-evaluated eligible Xpert MTB/RIF Ultra positive TB patients using the Xpert MTB/XDR platform regardless of Rifampicin resistance to identify additional drug resistance beside rifampicin. We analyzed patient demographics, characteristics, and the distribution of drug resistance drug-resistance categories descriptively. Of 509 TB patients identified, 70.9%(n = 360) were bacteriologically confirmed by Xpert MTB/RIF Ultra among whom 92.8%(n = 336) were eligible for reflex Xpert MTB/XDR testing. Of the eligible, 89.3% (n = 313) underwent reflex Xpert MTB/XDR testing. Results were successfully retrieved for 282 (90.1%). Overall, 8.5%(n = 24) of patients with XDR results had a form of drug-resistant tuberculosis. Mono-resistance to rifampicin (3.5%, n = 10) or isoniazid (3.2%, n = 9) was more prevalent than multi-drug-resistant tuberculosis (1.1%, n = 3), while only 0.4% (n = 1) had Pre-XDR TB. Non-rifampicin resistant drug-resistance, including isoniazid mono-resistance and isoniazid combined with fluoroquinolone or ethionamide resistance, were detected in 3.2% (n = 9) of all patients with XDR TB results (37.5% of all DR-TB cases). Reflex Xpert MTB/XDR testing enabled rapid identification of non-rifampicin-resistance drug-resistant TB cases that would have been missed by rifampicin-centric diagnostics. Strengthening the integration of MTB/XDR into routine diagnostic algorithms can improve early drug-resistant tuberculosis detection, guide individualized treatment, and prevent amplification of resistance in high-burden settings.

## Introduction

Zambia, one of the 30 high tuberculosis (TB) burden countries globally, continues to shoulder a significant TB disease burden with a high incidence of drug-resistance (DR). In 2024, the World Health Organisation (WHO) estimated Zambia’s total TB incidence at 272 per 100,000 population and that of multi-drug or rifampicin-resistant (MDR/RR) TB at 5.8 per 100 000 population [1]. Despite improvements in TB elimination efforts, such as increased DR TB surveillance through expanded GeneXpert MTB/RIF coverage countrywide, Zambia continues to face challenges in diagnosing DR TB. These challenges may potentially be contributing to a steady decline in DR TB notifications since 2018 [1]. According to the WHO estimates, about 1600 people developed MDR/RR TB in 2024, yet only 385 (24.1%) were confirmed in the laboratory [1] suggesting a large number of missing DR TB patients.

Like in other resource limited settings, the under-diagnosis of DR TB is attributable to limited access to advanced molecular diagnostics as well as challenges in sample transportation and laboratory network to facilitate culture and drug susceptibility testing in reference laboratories [2,3]. While the use of the GeneXpert Mycobacterium Tuberculosis/rifampicin (Xpert MTB/RIF) Ultra has improved rifampicin resistance detection, it does not detect any other resistance such as to first-line drugs including isoniazid and second-line DR such as ethionamide, fluoroquinolones and aminoglycosides such as kanamycin and amikacin [4]. This gap poses a significant risk: individuals with non-rifampicin resistance drug-resistant may be erroneously treated with first-line regimens, potentially leading to treatment failure, amplification of resistance, mortality and continued community transmission [5,6]. Therefore, rapid and comprehensive DR TB diagnostics are essential in high-burden settings.

The recently introduced WHO-recommended GeneXpert MTB/Extensively Drug-Resistant (XDR) assay builds upon the established Xpert MTB/RIF Ultra platforms to provide rapid, cartridge-based detection of resistance to isoniazid, fluoroquinolones, ethionamide and aminoglycosides (amikacin, kanamycin and capreomycin) within hours [7,8]. This tool has shown high sensitivity and specificity across diverse settings and is recommended for use in patients at risk of second-line DR TB or those with prior treatment history [9–11] and as a reflex test for all bacteriologically confirmed TB [4].

Although Zambia introduced reflex Xpert MTB/XDR testing in the programmatic setting in 2024, its uptake remains limited, and non-rifampicin resistant DR TB is still poorly characterized. In response, the United States Government funded Tuberculosis Local Organizations Network (TBLON) project supported the Ministry of Health (MoH) to conduct a targeted DR-TB active case finding (ACF) surge in May 2025. This was conducted across project-supported high TB burden sites that incorporated Reflex Xpert MTB/XDR testing for eligible bacteriologically confirmed TB patients. We report programmatic data from the surge to quantify the prevalence of non-rifampicin resistance drug-resistant TB among bacteriologically confirmed TB patients regardless of the rifampicin resistance status. The aim of this analysis was to describe the prevalence and distribution of drug resistance among bacteriologically confirmed TB patients subjected to reflex Xpert MTB/XDR testing.

## Materials and methods

### Study design and ACF surge setting

This was a retrospective analysis of routine programme data from a DR TB ACF surge. The surge was conducted between 5^th^ and 31^st^ May 2025. The surge was a targeted, multi-site surge activity conducted by Zambia’s National TB and Leprosy Programme (NTLP) with support from the TBLON project. It was implemented in 39 high-TB health facilities (all GeneXpert MTB/RIF sites) with a high TB case load in five of Zambia’s high TB burden provinces: Lusaka, Southern, Copperbelt, Northern and Central Provinces. Each participating province had specific Xpert MTB/XDR sites where samples were transported for reflex testing. Reflex Xpert MTB/XDR test is a follow on test conducted on all bacteriologically confirmed TB patients (excluding trace and regardless of the rifampicin resistance result) on residual specimens previously processed for Xpert MTB/RIF Ultra test or a new one if the initial sample was inadequate [12]. Provinces and facilities were selected based on their DR-TB burden: provinces with ≥25 DR TB notifications and health facilities with at least five [5] DR TB notifications in 2024 were included in the surge.

The participating health facilities routinely conduct DR TB surveillance activities through clinical screening and sample evaluation using GeneXpert MTB/RIF Ultra. Although reflex testing for all bacteriologically confirmed TB patients using Xpert MTB/XDR is required [13], it is not routinely available and performed. According to Zambia’s consolidated guidelines, all Xpert MTB/RIF positive TB patients (excluding trace) must undergo reflex Xpert XDR testing.

### Surge activity procedures

The DR TB ACF surge had a preparatory and an implementation phase.

#### Preparatory phase.

After co-developing the concept note with the NTLP, pre-surge key components included planning, logistics management, development of digital monitoring tools, health care workers capacity building as well as community mobilization and demand generation.

To ensure commodity availability, we conducted facility-level assessments to determine baseline stocks of GeneXpert MTB/RIF Ultra and Xpert MTB/XDR cartridges as well as TB drugs. Where necessary, MoH staff made emergency orders, and the project supported commodity transportation and distribution including redistributions. Furthermore, we developed a courier matrix to facilitate timely sample referral from participating facilities to Xpert MTB/XDR sites.

We developed a surge orientation package covering DR-TB definitions, case finding, integration of Xpert MTB/XDR testing, demand generation strategies and data entry procedures. Using this package, we capacity-built healthcare workers (HCWs) including clinicians, nurses, Community Health Workers (CBWs), and data staff through facility-based orientation sessions.

We implemented community mobilization and demand generation efforts throughout both the pre-surge and intra-surge periods. We developed a standardized community engagement script and applied it across interpersonal communication, media programs, and church visits to raise awareness of DR-TB, enhance service acceptability, and stimulate demand.

To facilitate adequate abstraction of information from registers and performance monitoring, the Strategic Information team developed an electronic data collection tool within REDCap version 14.8.2 for patient-level data management during the surge. The tool underwent iterative testing and revisions before rollout.

#### Implementation phase.

Three main categories of TB patients were targeted during this surge.

i. TB patients bacteriologically confirmed in the preceding two months using microscopy and urine LAM.
ii. Previously treated patients including smear positive sputum at month 2 of treatment/treatment after failure and treatment after loss to follow (≥2 continuous months of interrupted treatment) and TB relapse (previously treated for TB, declared cured or treatment completed at the end of their most recent course of treatment, and diagnosed with a recurrent episode of TB).
iii. Prospective bacteriologically confirmed TB patients diagnosed using Xpert MTB/RIF Ultra.

During the surge, patients under (i) and (ii) were evaluated (relapse) and re-evaluated (treatment after failure, treatment after lost to follow up) using Xpert MTB/RIF Ultra to determine rifampicin resistance and eligibility for reflex Xpert MTB/XDR testing. The Xpert MTB/RIF positive TB patients, from all categories (excluding trace results) were couriered to Xpert MTB/XDR sites for further evaluation to rapidly detect resistance to isoniazid, fluoroquinolones, ethionamide and aminoglycosides. Where initial samples submitted were inadequate, fresh ones were collected for Xpert MTB/XDR testing. Facilities without Xpert MTB/XDR platforms onsite couriered samples to other facilities with XDR machines and waited for receipt of results.

#### Surge monitoring.

Daily surge implementation was jointly monitored by TBLON and MoH teams through structured supervision visits that provided onsite support for data collection, quality assurance, and troubleshooting. Twice-weekly virtual performance review meetings were held to analyze trends, address barriers, and guide adaptive management. Furthermore, we disseminated weekly data summaries to facilities to reinforce accountability and enable timely intervention where challenges were noted.

#### Data collection and management.

During the surge, facility-level MoH staff routinely entered patient level data in the facility registers: TB Presumptive, TB treatment and laboratory registers. To collect the data relevant to this analysis, we developed, piloted and used a digitized data collection tool in REDCap v14.8.2. We abstracted de-identified data from the registers into the digitized tool between July and September 2025.

#### Data analysis.

Data was extracted from REDCap, cleaned and imported into STATA v17.0 (StataCorp, USA) for analysis. We conducted descriptive data analysis of the patients’ characteristics, levels of health facilities and types of drug resistance among bacteriologically confirmed TB and DR TB patients. We did not conduct any inferential statistical tests considering the small absolute numbers of DR-TB patients identified.

### Definition of terms in the context of this study

i. Reflex Xpert MTB/XDR testing – A follow on GeneXpert MTB/XDR test conducted on all bacteriologically confirmed TB patients on residual specimens previously processed for Xpert MTB/RIF Ultra test or a new one if the initial sample was inadequate [12].
ii. MDR - Resistance to both isoniazid and rifampicin with or without resistance to other anti TB drugs [14]
iii. Mono-resistance– Resistance to only one first line drug [15].
iv. Pre- extensively drug-resistant (Pre-XDR) TB – MTB that fulfils the definition of multidrug-resistant or rifampicin-resistant TB (MDR/RR-TB) and also resistant to any fluoroquinolone [16]
v. Poly-resistance - resistance to more than one first-line anti-TB drug, beside both rifampicin and isoniazid [14].
vi. All forms of Drug resistance: refers to different combinations of drug resistance such as isoniazid resistance combined with rifampicin/ethionamide/amikacin/fluoroquinolones e.t.c.

### Ethics statement

Ethics approval for the study was obtained from University of Zambia Biomedical Research and Ethics Committee, Lusaka, Zambia (Reference number 1773–2021) to analyse existing programme data. The University of Zambia Biomedical Research and Ethics Committee, Lusaka, Zambia (Reference number 1773–2021) also waived the requirement of written informed consent, as the analysis was retrospectively performed using routine data which was de-identified before analysis.

## Results

**Fig 1:**
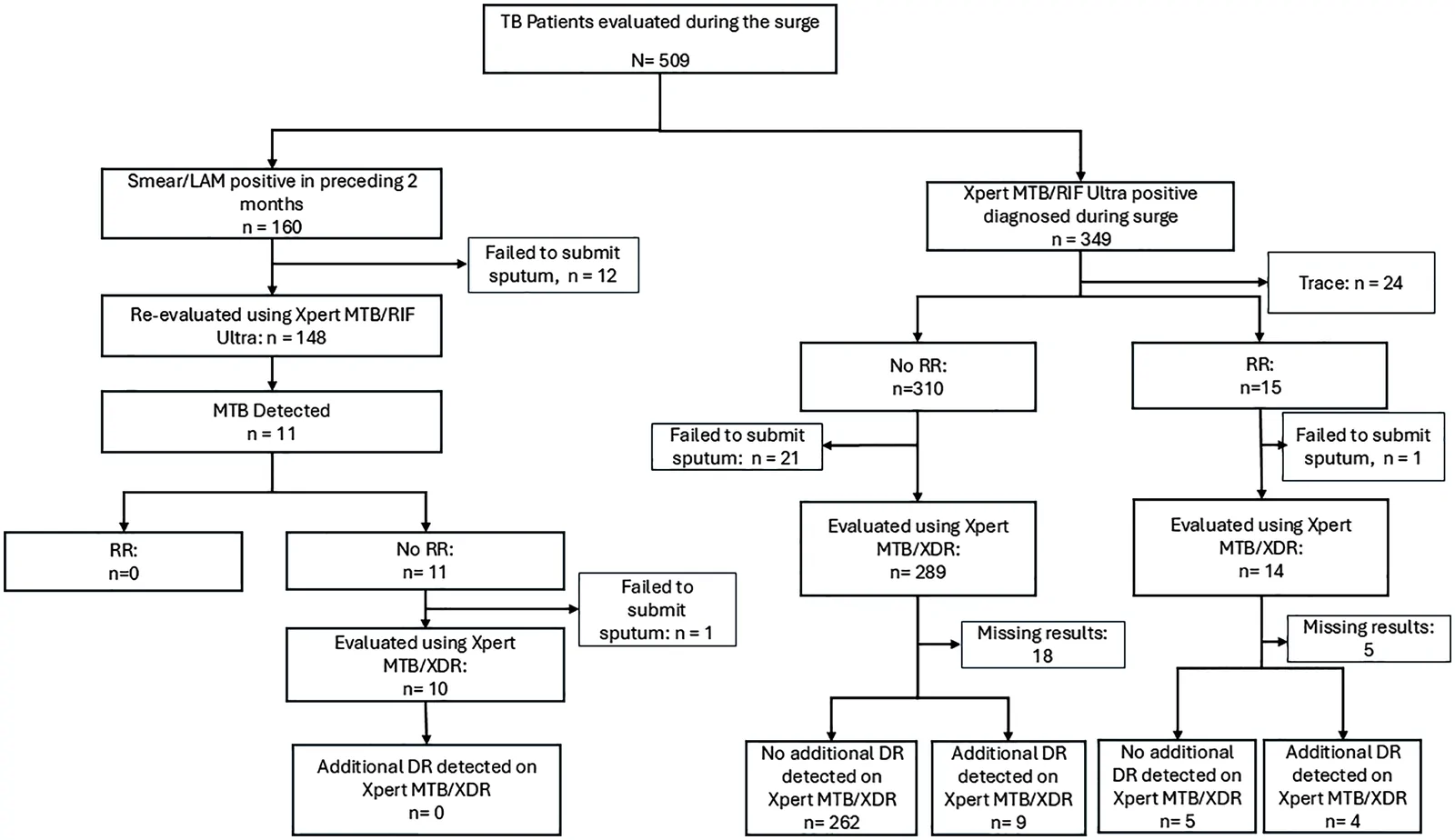
Patient flow chart.

We identified 509 TB patients (Fig 1): 160 (31.4%) were either previously diagnosed using smear microscopy/LAM without Xpert MTB/RIF tests or were smear positive at month 2 of treatment (category 1) and 349 (68.6%) were Xpert MTB/RIF positive diagnosed during surge period (Category 2). Among the 160 category 1 patients, 148 (92.5%) were re-evaluated using GeneXpert MTB/RIF during the surge with 11 (7.4%) testing positive for Mycobacterium tuberculosis (MTB). Among the 349 patients with Xpert MTB/RIF positive results, 15 (4.3%) had rifampicin resistant (RR] TB; 310 (88.8%) rifampicin susceptible and 24 (6.7%) had trace results. Among 15 patients with RR TB, 14 (93.3%) submitted samples for reflex Xpert MTB/XDR testing with 4 (28.6%) detected with additional DR. Of the 310 rifampicin susceptible patients, 289 (93.2%) submitted samples for reflex XDR testing to identify non-rifampicin resistant DR TB and results were successfully retrieved for 271 (93.4%) patients (a total of 282 results from category 1 and category 2). Overall, 24 (8.5%) of the 282 bacteriologically confirmed TB patients with accessible XDR results were found to have DR TB.

### Participant characteristics

Overall, 509 TB patients were identified during the surge (median age 38 years, IQR 29–47). The majority of the patients were males (70.9%, n = 361/506) and were aged ≥15 years (98.4%, n = 498/506) (Table 1). We identified patients from across all levels of healthcare with the highest representation from health centres (35.8%, n = 181/505) and first-level hospitals (34.1%, n = 172/505). The rest were from second-level hospitals (14.7%, n = 74/505), and third-level hospitals (15.4%, n = 78/506). About 35.9% (n = 180/501) of patients were HIV-positive, while only 3.6% (n = 18/501) had unknown HIV status.

**Table 1:** Characteristics of patients included in the study.

Variable	*n* (%)
Age, Median (IQR) *(N – 509)*	38 (29-47)
Age Group *(N - 506)* ^a^	
0-4	8 (1.6)
5-14	11 (2.2)
15+	487 (96.2)
Sex *(N- 506)* ^a^	
Male	361(70.9)
Female	148 (29.1)
Level of Health Facility *(N - 505)*^b^	
Health Centre	181 (35.8)
First Level Hospital	172 (34.1)
Second Level Hospital	74 (14.7)
Third Level Hospital	78 (15.4)
HIV Status *(N - 501)*^c^	
Positive	180 (35.9)
Negative	302 (60.3)
Unknown	18 (3.6)

^a^Three (3) missing values; ^b^Four (4) missing values; ^c^Eight (8) missing values.

### Prevalence and distribution of drug resistance among TB patients

While the overall prevalence of DR TB among all bacteriologically confirmed TB patients was 8.5% (n = 24/284) (75%; n = 18 being male), the prevalence of RR/MDR (all forms) was 5.3% (n = 15/284) and that of isoniazid (all forms) was 4.6% (n = 13/284) (Table 2). Only one patient (0.4%) had combined RR, isoniazid and fluoroquinolone resistance (Pre-XDR). For mono-resistance among patients with accessible XDR results, 3.5% (n = 10/284) and 3.2% (n = 9/284) were mono-resistant to rifampicin and isoniazid respectively. Additionally, among all bacteriologically confirmed drug-susceptible TB patients with XDR test results, 1.8% (n = 5/284) had resistance to fluoroquinolones.

**Table 2:** Drug resistance among bacteriologically confirmed TB patients.

Drug resistance among TB patients	*Total TB patients with drug resistance (n)*	% drug resistance among all Bact. Confirmed TB with XDR Results (*N = 282*)	% drug resistance among total DR-TB (*N* = 24)
RR + (INH or FQ) Resistance*	15	5.3	62.5
MDR (INH + RR)	3	1.1	12.5
INH + RR + FQ	1	0.4	4.2
Rif. mono-resistance	10	3.5	41.7
INH+ (RIF or ETH or FQ) Resistance	13	4.6	54.2
INH mono-resistance	9	3.2	37.5
INH + ETH	2	0.7	8.3
INH + FQ	1	0.4	4.2
FQ mono-resistance	5	1.8	—

RR – rifampicin resistance, INH – Isoniazid, Rif – Rifampicin, ETH – Ethionamide, FQ – Fluoroquinolone, *One (1) RR patient did not submit sputum for XDR testing.

Analysis of the distribution of drug resistance among DR TB patients (n = 24) showed that overall resistance to rifampicin (all forms) and isoniazid (all forms) was 62.5% (n = 15/24) and 54.2% (n = 13/24) respectively (Table 2). Other categories of resistance among DR-TB patients included rifampicin mono-resistance (41.7%, n = 10/24), isoniazid mono-resistance (37.5%, n = 9/24), combined rifampicin and isoniazid resistance (MDR-TB) (12.5%, n = 3/24), resistance to isoniazid and ethionamide (8.3%, n = 2/24), isoniazid and fluoroquinolone resistance (4.2%, n = 1/24), and triple resistance to isoniazid, rifampicin, and fluoroquinolone (4.2%, n = 1/24). In this study, we identified non-rifampicin resistance drug-resistant TB in 3.2% (n = 9/284) of all patients with XDR test results (37.5% of DR TB patients).

When stratified by patient category (Table 3), the prevalence of MDR/RR was higher among relapse cases (8.5%, n = 4/47) while isoniazid mono-resistance was higher among new TB patients (3.1%, n = 7/223) than among retreatment cases. Among new TB patients, only 0.4% (n = 1/223) had MDR-TB, while 3.5% (n = 8/223) had rifampicin mono-resistance, and 3.1% (n = 7/223) had isoniazid mono-resistance. Among TB relapse cases, 6.4% (n = 3/47) had MDR-TB, 4.3%(n = 2/47) rifampicin mono-resistance, and 2.1% (n = 1/47) isoniazid mono-resistance. The one patient categorized as treatment after failure had isoniazid mono-resistance while another patient who tested smear positive at two months of treatment remained susceptible to both rifampicin and isoniazid. All patients treated after loss to follow-up whose results were documented were susceptible to rifampicin and isoniazid with no other resistance detected.

**Table 3:** Drug resistance among bacteriologically confirmed TB patients by patient risk category.

			Distribution of drug resistance			
Patient Category	Total *(N)*	Susceptible to RIF + INH; *n* (%)	RIF + INH; *n* (%)	RIF + INH + FQ; *n* (%)	RIF mono resistance; *n* (%)	INH mono resistance; *n* (%)
Total Bact. Confirmed with XDR Results^a^	282	260 (92.2)	3 (1.1)	1 (0.4)	10 (3.5)	9 (3.2)
*New*	223	206 (92.4)	1 (0.4)	0 (0.0)	8 (3.6)	7 (3.1)
*Relapse*	47	39 (87.2)	2 (4.3)	1 (2.1)	2 (4.3)	1 (2.1)
*TAF*	1	0 (0.0)	0 (0.0)	0 (0.0)	0 (0.0)	1 (100.0)
*Treatment after LTFU* ^b^	8	4 (50.0)	0 (0.0)	0 (0.0)	0 (0.0)	0 (0.0)

^a^Four patients had undocumented RR results; ^b^Four patients had MTB undetectable results on the XDR platform hence no resistance profile indicated; LTFU – Lost to follow up; TAF – treatment after failure.

## Discussion

In this analysis of programmatic data, we report a DR TB prevalence of 8.5% among bacteriologically confirmed TB patients. Among the DR TB patients, a high proportion (62.5%) had RR detected on GeneXpert MTB/RIF and over one-third (37.5%) had other forms of resistance detected on GeneXpert MTB/XDR. The prevalence of any isoniazid resistance among patients with accessible XDR test results was 4.6%. One pre-XDR TB patient was diagnosed representing 0.4% of bacteriologically confirmed TB patients with XDR results and 4.2% of the DR TB patients. Of all drug resistance detected, 79.2% had mono-resistance (either rifampicin or isoniazid). Mono-resistance to either rifampicin or isoniazid was 5 times more prevalent than MDR-TB in this study.

These findings are similar to the results of Zambia’s most recent DR TB survey where MDR was 8 and 3 times less common than rifampicin and isoniazid-resistance respectively [17]. While the national DR-TB survey reported a higher prevalence of isoniazid resistance (3.9%) compared to RIF resistance (1.5%), our study found comparable rates of rifampicin mono-resistance (3.5%) and isoniazid mono-resistance (3.2%) among TB patients whose XDR test results were accessible. Our study found a lower overall prevalence of drug resistance compared to global estimates of 11.6%, 15.7% and 9.4% for MDR-TB, isoniazid-mono-resistance and rifampicin mono-resistance respectively [18]. Despite this, results align with regional and global observations suggesting that mono-resistance remains a significant, and sometimes underestimated, component of the DR-TB burden [18–20].

We also found that over one-third of all DR TB patients detected in this surge had non-RIF resistant DR TB: mainly isoniazid with or without fluoroquinolone and ethionamide resistance. While the high prevalence of non-rifampicin resistant DR TB observed in our study among DR TB patients contrasts with the widely held assumption that rifampicin resistance serves as a reliable proxy for concurrent isoniazid resistance; and thus MDR-TB [21], it aligns with emerging global evidence indicating that a substantial proportion of DR TB cases are rifampicin-susceptible but resistant to other anti-TB drugs. For example, a multi-country review of 211,753 patients from 156 countries globally established a isoniazid mono-resistance prevalence of 8.4% among all TB patients [22]. Similarly, a systematic review and meta-analysis with a sample size of 318,430 participants established a pooled isoniazid resistance prevalence of 15.7%; higher than MDR (11.6%) and rifampicin (9.4%) resistance [18]. It is, therefore, possible that TB patients with non-rifampicin resistant DR TB such as isoniazid mono-resistance may often be missed in rifampicin-centric testing algorithms, hence misclassified and notified as DS TB. This misclassification leads to missed cases of DR TB, thereby undermining DR TB case-finding efforts. In addition, mis-classified patients are often inappropriately started on isoniazid based first-line regimens. Considering isoniazid resistance requires a tailored treatment regimen with inclusion of levofloxacin [21], mis-classification results in suboptimal therapy and potentially poor treatment outcomes such as treatment failure, amplification of resistance especially where fluoroquinolone resistance is also missed [22–26]. Misclassified isoniazid resistance can, therefore, delay timely initiation of appropriate treatment, slow down the interruption of community TB transmission and compromise treatment outcomes even when rifampicin is established to be effective [27].

Findings from this multi-site implementation suggests operational feasibility of reflex Xpert MTB/XDR testing within routine programmatic conditions in Zambia. Over one-third of bacteriologically confirmed TB patients in this study were identified at primary health care (PHC) facilities, which aligns with existing evidence that nearly half of all TB cases in Zambia are diagnosed at the PHC level [28]. This demonstrates the feasibility of rolling out Xpert MTB/XDR in PHC where access to LPA as well as culture and phenotypic DST (pDST) are often limited in low and middle income countries like Zambia [29]. While culture-based phenotypic DST has been useful and remains the gold standard for detecting non-rifampicin resistant DR TB in Zambia and many other countries [30], poor access especially in PHC settings, characterized by longer turnaround time associated with sample courier to centralized laboratories and results transmission back to referring facilities, delays decision-making on the choice of appropriate treatment regimen [31–33]. A substantially reduced time to accurate diagnosis achieved through decentralized DST using platforms like Xpert/XDR testing; and prompt initiation of appropriate treatment is, therefore, a principle benefit for both the healthcare workers and TB patients [34]. Furthermore, The high proportion of non-rifampicin-resistant DR-TB diagnosed at PHC level through reflex XDR testing highlight the utility of the test in a algorithm that is not rifampicin resistance focused.

Although Xpert MTB/XDR assays demonstrate slightly lower sensitivity for detecting ethionamide and FQ resistance compared to pDST and may fail to capture hetero-resistance [35], they remain valuable for immediate patient management in programmatic settings. Within diagnostic algorithms, Xpert MTB/XDR testing must, therefore, be strategically positioned in near point-of-care settings as a follow-on test for individuals with bacteriologically confirmed TB. This would facilitate quick decision making as results are obtainable within 90 minutes [31]. Overall, Zambia’s existing GeneXpert network, combined with demonstrated programmatic experience, provides a strong foundation for national scale-up of reflex Xpert MTB/XDR testing.

Besides deployment of XDR platforms, utilization must be optimized to get the desired benefit. Consistent with the surge approaches, strengthening health systems including staff capacity building, improved laboratory logistics, efficient sample transportation networks, robust monitoring and data management systems, and enhanced ownership among healthcare workers, including laboratory personnel, is essential to optimize the utilization of reflex Xpert MTB/XDR testing [34]. National TB programmes should, therefore, prioritize capacity building of healthcare workers, strengthening of laboratory systems, robust monitoring and data management, and fostering local ownership to optimize the utilization of reflex Xpert MTB/XDR testing.

Despite not conducting a full cost or cost-effectiveness analysis, the implementation of reflex Xpert MTB/XDR testing in Zambia is likely to be cost-effective when considered from a health system’s perspective. Although upfront costs related to equipment utilization, staff capacity building, technical support, and sample courier to XDR sites are higher than routine rifampicin-focused testing, these are likely to be offset by downstream savings from improved treatment targeting, reduced diagnostic delays, reduced cost on sample transportation, prevention of inappropriate first-line therapy, averting of treatment failure, reduced amplification of resistance, and minimize costly retreatment, hospitalization and mortality [18,22]. Furthermore, decentralized molecular testing reduces reliance on culture and phenotypic drug susceptibility testing, which are resource-intensive and associated with longer turnaround times [29–31]. Economic modelling studies in similar high-burden settings have demonstrated that rapid molecular DST platforms become increasingly cost-effective when integrated into existing GeneXpert networks and applied at scale, particularly where DR-TB is underdiagnosed [31,34]. Collectively, these factors suggest that scaling reflex Xpert MTB/XDR within Zambia’s existing diagnostic infrastructure represents a high-value investment aligned with universal access to drug susceptibility testing.

This study has several notable limitations and strengths. The short implementation period limited the number of DR TB cases detected, constraining precision in estimating less frequent resistance categories and reducing the scope of analyses that could be conducted. Its focus on urban and peri-urban facilities may not fully capture rural diagnostic realities, while reliance on routine programmatic data could have introduced reporting gaps. Additionally, confirmatory phenotypic DST was not performed. Another limitation was that we did not conduct a full cost or cost-effectiveness analysis in this paper. Despite these limitations, the study provides timely, operationally relevant evidence supporting national scale-up of Xpert MTB/XDR testing to enhance rapid detection and management of DR tuberculosis beyond rifampicin resistance. It represents one of the first programmatic evaluations of the Xpert MTB/XDR assay in Zambia and the region, conducted under real-world MoH conditions across 39 high-burden facilities in five provinces, making the findings both programmatically relevant and generalizable to the rest of the country and potentially other similar contexts. Integration of digital monitoring tools, logistics support, and staff capacity building further strengthened implementation quality to consider when integrating reflex Xpert MTB/XDR testing into routine diagnostic algorithms.

## Conclusion

The Xpert MTB/XDR assay proved to be a valuable addition to Zambia’s TB diagnostic tools during this nationwide MDR-TB surge with high utility at primary health care level facilities. The ability to detect non-rifampicin resistant DR TB rapidly is essential for accelerating early detection of DR TB, finding missed cases, achieving universal access to DST, optimizing treatment regimens, and improving TB elimination efforts at lower levels of care. We recommend a national scale-up of reflex Xpert MTB/XDR testing, integrated with robust supply chain management for laboratory commodities, health care workers training, and real-time data use, to advance the fight against DR-TB in Zambia and similar settings.

## Supporting information

S1 Data Dataset for the MDR TB surge. (XLSX)
